# A Few Remarks on Cholera

**Published:** 1854-10

**Authors:** 


We have received the following from a correspondent in reply to some editorial remarks in our last. EDS.

*A few Remarks on Cholera.* By CLERICUS, JR.

In the September number of your valuable periodical, I see in the editorial some remarks on the treatment of Cholera in Buffalo, and if you will allow me to speak bluntly, the ideas of treatment there advanced are gloriously indefinite and uncertain.

1st. Strychnine is condemned; to which I reply heartily, amen.

2d. John Bull's remedy, sulphuric acid, is "damned with faint praise." That remedy was probably suggested by the reading of Braithwaite's Retrospect, which, in my opinion, is enough to consign it to oblivion. Though I confess that I read Braithwaite, I must say that I have been sorely disappointed in the use of remedies therein recommended. It is mostly occupied by fanciful theories upon dry, occult subjects by lecturers, men whose business it is to talk and to write, in order to secure a reputation for authorship. So let sulph. acid go.

4th. Calomel and morphine in combination, are suggested. I dislike the union. In my limited experience opium is of little use in cholera. It exerts but little influence upon the discharges; merely diminishing their frequency but not changing their character. It assists congestion, a thing so much dreaded, and I think, brings on that typhus state which is as surely fatal as the collapse of cholera.

4th. You remark that no one has used calomel alone. Please to put down my name as one who has trusted to calomel, unadulterated, undiluted, unmixed with any other vile drug. I must, however, say that my experience in cholera is not large, but to me, thus far very instructive. Let me give my last case to illustrate its use:

*Sept.* 4, 1854. I was called to see an Irish emigrant with cholera. He was taken on the cars at 3, A. M., and I saw him at 3, P. M., of the same day. As I entered the room he was vomiting rice water, which was immediately followed by a discharge of the same character, which nearly filled an ordinary vessel; for the hour previous they had come on at intervals of ten minutes. He was cramping violently; the muscles of the leg and thigh were involved; his countenance was dull, blue, haggard; tongue slightly cool; pulse small, rapid and feeble; voice hoarse, hands shriveled, and extremities very cold. I gave him 20 grs. dry calomel and a small piece of ice to assist him in swallowing it. Ordered bottles of hot water to be placed around him, (which, by the way, was not done.) The next morning I gave him 5 grs. more of calomel. He had not a single evacuation from his bowels until twenty-four hours after taking the calomel. He then had two slate-colored discharges. Reaction was fully established in three hours after taking the calomel. The cramps ceased in one hour and twenty minutes after taking it.

The third day I gave him a dose of castor oil and brought away an immense dark greenish-black mass, resembling a dose of black cats well stewed. As he had some pain after this discharge, I gave him a very small portion of morphia to allay the present pain. Here is a case treated with calomel alone, no stimulants of any kind were used. The man was confined to his bed four days. I could point out to two other cases, as well marked as the above, where the same treatment was pursued with like success. I have had four cases of well-developed cholera. Three of these got well. The fourth was a man of very dissipated habits, had labored under diarrhœa for a week: he died four hours after treatment.

I have had twelve cases of choleric diarrhœa, by which I mean a painless profuse diarrhœa, attended with nausea, great prostration, the discharges containing no bile, but varying from a pale drab to a milk color, and even pure rice water, all of which yielded to calomel without opium. One cathartic dose of calomel usually did the business. In all cases it arrested the discharges quicker and surer than any combination of opiat s or astringents that I have hitherto employed. Such is my experience, and you are welcome to it in full. The hurry of business does not admit such a nicety of observation as I could wish. Calomel is my sheet anchor; all other remedies merely adjuvants. Dry heat, by means of hot bottles or bricks, no rubbing, no dosing, no stimulants. I have seen several fatal cases of cholera, here, but they were under the care of other physicians previous to my attendance, and they died under stimulants: common salt, Cayenne pepper, frictions that would make a horse howl with pain.

I hope that you will try this remedy alone in Buffalo, adding merely dry heat. After the two doses above let the patient alone. By the way, I repeat the calomel, if vomited, till enough is retained to suit me.

